# First record of the marine turtle leech (*Ozobranchus margoi*) on hawksbill turtles (*Eretmochelys imbricata*) in the inner granitic Seychelles

**DOI:** 10.4102/ojvr.v85i1.1604

**Published:** 2018-08-30

**Authors:** Byron M. Göpper, Nina M. Voogt, Andre Ganswindt

**Affiliations:** 1 Department of Anatomy and Physiology, University of Pretoria, South Africa; 2 Mammal Research Institute, Department of Zoology and Entomology, University of Pretoria, South Africa

## Abstract

Ozobranchus spp. are leeches that feed solely on turtle blood. They are common ectoparasites found on a range of marine turtle species, with some species of the leech being implicated as vectors of fibropapilloma-associated turtle herpesvirus (FPTHV). Green (*Chelonia mydas*) and hawksbill (*Eretmochelys imbricata*) turtles are the two commonly occurring species in the inner granitic islands of the Seychelles. Routine monitoring of nesting turtles on Cousine Island, Seychelles, allowed for opportunistic sightings of leeches on two hawksbill females. In both cases infestation was low, with three leeches collected off one female turtle and five off the other. No obvious signs of papillomas secondary to infection of FPTHV were seen. All of the turtle leeches collected were determined to be *Ozobranchus margoi* as they had five pairs of lateral digiform branchiae. The specimens were deposited in the Seychelles Natural History Museum on Mahé. To the best of our knowledge this is the first record of *Ozobranchus margoi* recorded in the inner granitic Seychelles on hawksbill turtles.

## Introduction

The genus *Ozobranchus* is characterised, among other features, by their anterior abdominal somites that each possess a pair of gills that are divided distally into many branchiae (Richardson 1969). They are the only non-piscicolid marine leeches to be permanent parasites of sea turtles and attach onto the turtle’s skin, particularly around the cloaca, head region and flippers (Sawyer 1986). Ozobranchs are stationary leeches, remaining on their host for their entire life cycle. Numerous cocoons are deposited on the turtle’s plastron where they hatch. Newly hatched leeches feed on the same host as their parents. This cycle is repeated, ultimately leading to large numbers of leeches on an individual (Sawyer 1986). Owing to the difficulties of studying their sea turtle hosts, very little is known about sea turtle leeches and whether they are able to survive partly without a host or utilise an alternate host (McGowin et al. 2011). However, more knowledge about the life cycle of sea turtle leeches would be beneficial, as some species of *Ozobranchus* have been implicated as mechanical vectors of fibropapilloma-associated turtle herpesvirus (FPTHV), a neoplastic disease causing epithelial tumours in sea turtles (Greenblatt et al. 2004).

*Ozobranchus margoi* parasitise several species of sea turtle, namely green turtles (*Chelonia mydas*; Richardson 1969), Kemp’s ridley turtles (*Lepidochelys kempii*; Davies & Chapman 1974), hawksbill turtles (*Eretmochelys imbricata*; Bunkley-Williams et al. 2008), as well as loggerhead turtles (*Caretta caretta*; Insacco, Violani & Zava 2000). However, *O. margoi* show some degree of host preference and are most frequently associated with loggerhead turtles (Bunkley-Williams et al. 2008).

Geographically there have been reports of *O. margoi* from Florida (Davies 1978; Davies & Chapman 1974; McGowin et al. 2011; Sawyer, Lawler & Oversrteet 1975; Truong 2014; Truong & McGowin 2011), North Carolina (Schwartz 1974), Hawaii (Balazs 1980), Barbados (Truong 2014), Brazil (Peralta et al. 2003; Rodenbusch et al. 2012; Truong 2014), Puerto Rico (Bunkley-Williams et al. 2008), Uruguay (Cordero 1929), Adriatic Sea (Piccolo & Manfredi 2003; Scaravelli, Affronte & Costa 2003), Italy (Apathy 1890), Mediterranean sea (Insacco et al. 2000), Tunisia (Karaa et al. 2011), South Africa (Hughes, Bass & Mentis 1967), India (Sanjeeva Raj 1959), Japan (Oka 1927), Taiwan (Cheng-Tsung & I-Jiunn 2013; Tseng, Leu & Cheng 2017) and Australia (Loop, Miller & Limpus 1995; Richardson 1969). However, there are no published reports documenting the occurrence of *O. margoi* on turtles in the inner granitic Seychelles. To the best of the authors’ knowledge, this is the first recorded report of *O. margoi* parasitising *E. imbricata* in the Seychelles Archipelago.

## Material and methods

Observations were made on Cousine Island (-4.350577°S, 55.647527°E), a 25-hectare island with a 1-km stretch of beach. The island is situated in the inner granitic Seychelles, Seychelles Archipelago, Indian Ocean. Within the inner granitic islands, Cousine Island is an important nesting beach for hawksbill turtles (*E. imbricata*). The islands’ conservation and turtle monitoring programme has been running since 1991 (Hitchins et al. 1999). While following Cousine Island’s standard routine monitoring protocols set out for nesting female hawksbill turtles, leeches were randomly spotted and opportunistically collected from two separate nesting female hawksbill turtles that came up to nest on Cousine Island’s beach during the 2015–2016 season. Collection of specimens took place on 12 January 2016 and 22 January 2016.

## Results

**FIGURE 1:**
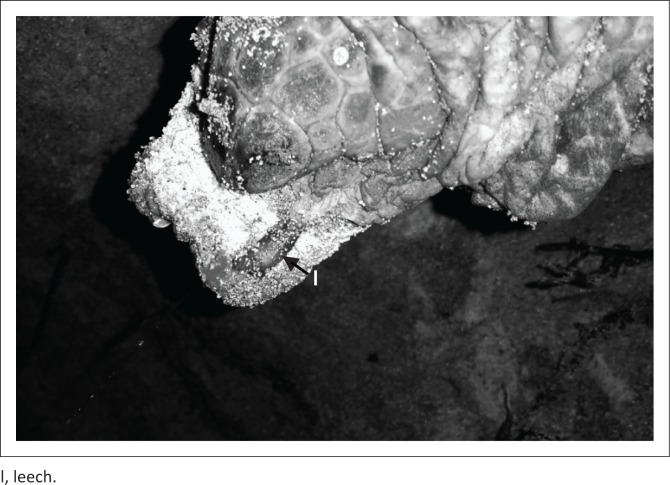
*Ozobranchus margoi* observed on nesting hawksbill turtle. Photo taken of tail and cloaca while eggs were being laid.

All leeches were located attached to the soft tissue around the cloaca of both female turtles (Figure 1). A total of eight leeches were collected, three off the female turtle with flipper tags SCA 3814 (left) and SCA 3815 (right), and five off the female turtle with flipper tags SCA 7048 (left) and SCA 7668 (right). Five specimens were preserved in 70% ethanol and the remaining three in 10% formalin. Subsequently, three of the specimens were deposited at the Natural History Museum, Mahé, Seychelles, for further studies and to make them available to other researchers. Therefore, the specimens’ corresponding voucher numbers are: 1443/16 (collection date: 12 January 2016) preserved in 70% ethanol, 1444/16 (collection date: 22 January 2016) preserved in 70% ethanol and 1445/16 (collection date: 22 January 2016) preserved in 10% formalin.

**FIGURE 2:**
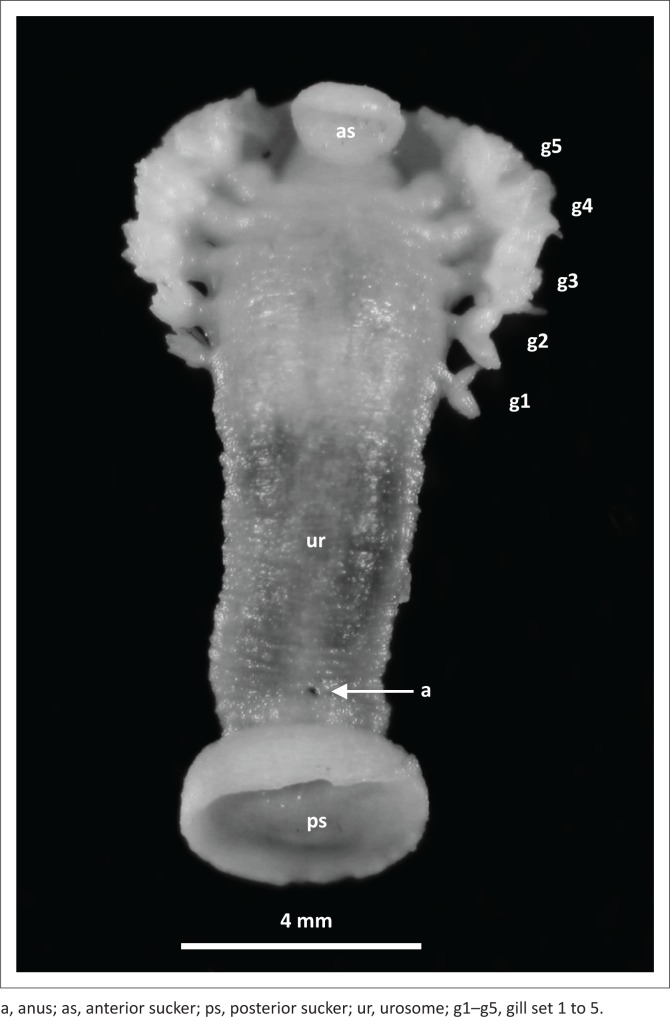
*Ozobranchus margoi* specimen.

The bodies of all eight specimens showed a distinguishable trachelosome and urosome, with a posterior sucker clearly visible. Furthermore, five pairs of gills were visible on the urosome (Figure 2). Based on these characteristics, the leeches were identified as *Ozobranchus margoi* (Davies 1978).

## Discussion

Based on a literature search for published cases of *O. margoi* using various keyword combination searches in Google Scholar and the database on the *Sea Turtle Network* (http://www.seaturtle.org/library/) no records of *O. margoi* parasitising *E. imbricata* in the Seychelles could be found. Samways et al. (2010), the main reference text for Cousine Island’s fauna, flora and ecology, also revealed no records; therefore, to the best of the authors’ knowledge, this is the first record of *O. margoi* parasitising on hawksbill turtles in the inner granitic Seychelles.

Green and hawksbill turtles are the two commonly occurring species in the inner granitic islands of the Seychelles. Future standard operating procedures for monitoring protocols of nesting female turtles are encouraged to include the recording of any observations of leeches or fibropapillomas. No obvious signs of papillomas were seen on the turtles in this study and in both cases infestation of leeches was low. As leeches are common on green turtles in other regions of the world, it would be interesting to further investigate the occurrence of *Ozobranchus* spp. in the Seychelles sea turtle populations, especially to see if other species (e.g. *O. branchiatus*) are also present.
